# Binge-watching Uncovered: Examining the interplay of perceived usefulness, habit, and regret in continuous viewing

**DOI:** 10.1016/j.heliyon.2024.e27848

**Published:** 2024-03-13

**Authors:** Maria Bastos, Mijail Naranjo-Zolotov, Manuela Aparício

**Affiliations:** NOVA Information Management School (NOVA IMS), Universidade NOVA de Lisboa, Campus de Campolide, 1070-312 Lisboa, Portugal

**Keywords:** Binge-watching, Continuous intention, Engaging behavior, Habit, Regret, Social influence, Netflix, TV streaming

## Abstract

Binge-watching has become one of the most popular ways for people to spend their free time. Binge-watching refers to watching more than two episodes of a television show in a single sitting. This pattern of behavior can be seen in people of a wide range of ages, but it is particularly widespread among people of millennial age and younger. In this study, we propose a model that explains binge-watching engagement by theorizing and testing the association of social influence on perceived usefulness, regret on the continuous intention of binge-watching, and habit on continuous intention and binge-watching engagement. The authors evaluated the model using data collected from 225 respondents. The results supported the proposed hypotheses and confirmed that regret does not neutralize the positive – and strong – effect of perceived usefulness and habit on binge-watching.

## 1 Introduction

Binge-watching consists of watching several episodes of a TV series in just one sitting [1]. A survey made in the United States in 2019 showed that more than 50% of adults under the age of 45 reported binge-watching [2]. Our society is becoming increasingly dependent on technology [3], and not only work time but also leisure time is spent in front of a screen. People are spending more time in front of a screen than ever before – a behavior that, in the long term, may seriously jeopardize the well-being of individuals and society.

Netflix pioneered the phenomenon of binge-watching with its subscription-based TV streaming services and video-on-demand (VOD) offerings. The platform revolutionized content consumption in 2013 when it began releasing entire seasons of shows at once, enabling viewers to watch entire seasons in just a few days or even one day [4,5]. Matrix [6] noted the growing association between the terms binge-watching and Netflix, while Sung et al. [7] identified Netflix as a catalyst for the trend. Although binge consumption of content may have existed previously, today's binge-watching experience offers viewers greater control over their content choices [1]. Netflix facilitates binge-watching through strategies such as releasing entire seasons, omitting commercials, auto-playing subsequent episodes, and utilizing sophisticated recommender systems and algorithms [5].

Although the definition of binge-watching is not consistent across the literature, for this study, we adopt the definition of binge-watching, which refers to watching more than two episodes of a television show in a single sitting [1]. We consider this definition as the most objective when trying to distinguish binge-watchers from non-binge-watchers. Moreover, this definition draws a line that divides the previous technology, where the user had no control over the number of episodes to watch, and the current TV streaming services and VOD. Other studies have expanded this definition, considering several factors such as age, occupation, and family situation [5,8]. For instance, a working person might consider watching two episodes as binge-watching if the time for watching TV is restricted, but perhaps a student who might enjoy more free time and fewer responsibilities could consider watching those two episodes as something lighter than binge-watching. Sung et al. [7] incorporated three additional elements: amount of time, frequency, and level of immersion in the story. The amount of time spent watching is crucial to consider, as individuals may watch varying numbers of episodes depending on the program length.

Horeck et al. [9] defend that there are two constant factors that pertain to binge-watching: the fact that it is the viewer who decides when and what to watch and that it has to do with only serialized programming. Because it is such a recent phenomenon, only a few studies have been conducted to explore and identify the motivations behind binge-watching behavior [10,11], its contextual factors [12], and the influence of personal traits [13,14]. However, the associations between the perception of binge-watching as harmless entertainment, habit, and regret with the continuous intention to binge-watch remain unexplored. Consequently, the present study contributes to shed light on the understanding of binge-watching intention and continuous intention, as well as the impact on binge-watching engagement. Here, we propose a theoretical model and test the influence of these variables with an empirical study using structural equation modeling (SEM) based on data collected from 225 binge-watchers. This study extends our knowledge of the binge-watching phenomenon by investigating elements that are likely to influence an individual's intention to continue binge-watching and engaging behavior.

This study offers three main contributions to the understanding of binge-watching behavior. First, it builds upon technology adoption theory to investigate the drivers of continuous intention and engagement in binge-watching, thereby expanding the limited literature on this widespread phenomenon. Second, the research demonstrates that both habit and continuous intention significantly influence binge-watching engagement, providing new insights into the complex interplay between these factors. Lastly, the study explores the negative association between feelings of regret after binge-watching and continuous intention, showing that these feelings can reduce the intention to continue binge-watching without necessarily stopping the behavior altogether.

## 2 Consequences of binge-watching

The literature examined records of real-world video-on-demand of binge-watching viewers, constructing a statistical viewership model that infers user features based on the number of episodes watched and identifying different types of binge behavior (binge and hyper binge) and found that binge-watchers are more likely to view content out of order [15]. However, this behavior is primarily dependent on the show's content and is more common in shows without a dominant storyline across episodes, suggesting that binge-watching is more prevalent when viewing comedy shows rather than dramas. In contrast, there are other studies that suggest that dramas are the most binge-watched shows due to their ongoing storylines that entice viewers to keep watching [7]. These studies also found that binge-watching is more likely to occur on weekends rather than weekdays, with TVs being the dominant viewing device over tablets and smartphones.

Viewers often multitask during binge-watching sessions, engaging in activities such as eating or checking their mobile phones [12,16]. These distractions appear to impact users' immersion in the story, with engagement in other activities increasing after an average of three to four episodes, leading to a loss of focus on the story's details. Binge-watching sessions also frequently result in viewers postponing planned activities, such as chores, school assignments, work tasks, sports, or social events. Postponing these activities often triggers feelings of guilt [16] or even reduced happiness after binge-watching [12]. However, other studies have found contradictory evidence, suggesting that binge-watching may lead to positive outcomes when planned, social, and attentive [17]. Furthermore, De Keere et al. [18] suggest that binge-watching is fun and can even be controlled by some types of viewers.

Binge-watching may be also associated with psychological effects. For instance, Ahmed [19] found out that binge-watchers show higher symptoms of depression, being this association stronger for females, implying that depression may be a trigger by feelings of depression. However, although binge-watching may be correlated with depression, it was not found related to loneliness. Another suggested that negative impacts can also involve health and social effects, regret of feeling of guilt and lack of control when there is an excess in binge-watching [20].

The literature further explores the influence of personal traits on binge-watching. For instance, the association between individual differences, such as the need for cognition and sensation seeking, and binge-watching, and how these traits moderate the effects of binge-watching motivations on actual binge-watching behavior. These personal traits positively correlate with binge-watching and play an integral role in moderating specific binge-watching behaviors [14]. Other studies also examined psychological traits in relation to binge-watching behavior, finding that individuals who seek immediate gratification tend to engage more strongly in binge-watching than those who prefer delayed, more attractive rewards [21].

Both abovementioned studies determined that the psychological trait of need for cognition strongly influences binge-watching behavior. This suggests that individuals with higher levels of curiosity are more likely to binge-watch and consume entire series quickly in order to satisfy their curiosity immediately.

## 3 Hypotheses development and theoretical model

### 3.1 Social influence and its impact on binge-watching decisions

Venkatesh and Davis [22] demonstrated that social influence in technology usage can indirectly affect individuals through perceived usefulness, a concept applicable to the binge-watching context. This occurs when one's intention to continue binge-watching is influenced by others' perceptions of the activity's usefulness. Consequently, social influence is linked to one's perceived usefulness of technology. Prior research has investigated the impact of descriptive social norms (what most people typically do) on perceived usefulness in the context of fitness app usage, with significant results [23]. In the case of binge-watching, individuals exposed to the idea that binge-watching is an effective and enjoyable form of entertainment may internalize this notion, influencing their own perceptions of the activity's usefulness. This, in turn, affects their intention to continue binge-watching as a means of feeling connected with others (identification).

Moreover, when users share their positive experiences with others, they contribute to the establishment of a social norm that reinforces the perceived usefulness of binge-watching. This social influence further strengthens the association between perceived usefulness and the continuous intention to binge-watch, as users seek to maintain social integration and identify with their peers who also engage in binge-watching. As corroborated by other studies [[23], [24], [25]], it is reasonable to assert that social influence has a direct effect on one's perceived usefulness of binge-watching. Therefore, we hypothesize.

H1 Social Influence will have a positive effect on an individual's perceived usefulness.

### 3.2 The roles of perceived usefulness and continuance intention in binge-watching

Perceived usefulness, initially proposed as a key determinant of information systems acceptance [26,27], refers in this study to the degree to which consumers believe binge-watching serves as a source of entertainment and enjoyment. When users perceive binge-watching as useful, they believe that it effectively fulfills its purpose of providing enjoyment and entertainment. This positive perception enhances their overall satisfaction with the activity and creates a sense of gratification. As a result, users are more likely to engage in binge-watching because they associate it with positive experiences and outcomes. The continuous intention to binge-watch may be driven by the user's belief that the activity will continue to offer the desired level of entertainment and gratification in the future. This intention may be reinforced by the user's positive experiences and the perceived usefulness of binge-watching, creating a self-reinforcing cycle of engagement. Previous studies have proven that there is a positive connection between satisfaction and behavioral involvement [8]. Consequently.

H2 Perceived usefulness is positively related to one's continuous intention to binge-watch.

H3 One's continuous intention of binge-watching is associated with engagement in binge-watching.

### 3.3 The impact of regret on binge-watching: negative consequences and continuous intention

In the fields of psychology, Zeelenberg and Pieters [28] define regret as “*the emotion that we experience when realizing or imagining that our current situation would have been better if only we had decided differently.”* Regret is a consequential emotion with a negative impact on people's well-being and is unique in its relation to decision-making. Individuals experience regret only if they could have prevented the present situation by making a different decision in the past, setting it apart from other negative emotions that do not require decision-making [29,30].

People who invest excessive time in binge-watching may feel regret for doing so. Binge-watching is akin to "heavy" traditional television viewing, as both primarily rely on consecutive content viewing. This excessive consumption is associated with numerous adverse consequences, such as reduced sleep, diminished outdoor activity, increased obesity risk, heightened anxiety, and lower life satisfaction [[31], [32], [33], [34]].

A study on binge-watching among college students aged 18–25 revealed some perceived consequences of the behavior, including impacts on physical and mental health as well as social life [35]. Some participants noted that binge-watchers might feel regret for the time taken from responsibilities and the potential role in developing mental health challenges. Using binge-watching as an escape or procrastination tool compounds stress as tasks remain uncompleted. Social interactions and relationships were also negatively affected, as binge-watching led to more limited human interaction and increased distance from friends and family. Communication and social skills appeared to suffer, as binge-watchers were perceived as more socially awkward. However, some students believed that binge-watching could serve as a catalyst for new friendships, offering opportunities to share popular jokes or even watch together [36]. Nevertheless, based on existing literature, the feeling of regret after binge-watching sessions is likely to negatively influence one's intention to continue the behavior. The following hypothesis is proposed.

H4 Regret is negatively associated with one's intention to continue binge-watching.

### 3.4 Habit formation and binge-watching behavior

Habits tend to form and become automatic over time as behaviors are frequently performed [37,38]. Establishing a habit requires a certain amount of repeated behavior [37]. Stable environments, characterized by the presence of similar situational cues and goals in regularly occurring situations, facilitate the performance of repeated behaviors with minimal cognitive monitoring [37,39]. In contrast, an unstable environment demands increased attention, as it presents new situations that require thought and consideration.

Once a habit is formed, there is a tendency to perform that behavior automatically [37,39]. E-commerce literature suggests that frequency habit positively impacts continuous intention to use [40]. Individuals who frequently watch TV shows may develop the habit of binge-watching. According to Venkatesh et al. [41], behaviors can be triggered by stimulus cues, which can activate both intentions and actions. This habit can evolve under specific contexts and circumstances, such as the time of day, the room in the house, or the presence of others who typically engage in the activity together. These factors can serve as environmental cues that might trigger both the intention to engage in the behavior and the behavior itself. Consequently, we hypothesize.

H5 Habit is positively related to one's continuous intention of binge-watching.

H6 Habit is positively related to one's binge-watching behavior.

**Fig. 1:**
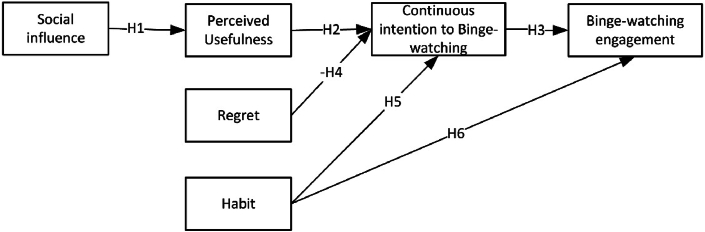
Binge-watching engagement theoretical model.

Based on the hypotheses proposed above, the theoretical model is presented in Fig. 1.

## 4 Methodology

### 4.1 Questionnaire design

To test the abovementioned hypotheses, we adapted measures from previous research on the constructs operationalized in the present theoretical model. With the adapted measures, we built a closed-ended questionnaire in the Qualtrics 3.0 survey tool. The electronic questionnaire was administered in a European non-English speaking country. The questions were initially prepared in English, translated to the original country language, and then back-translated to English to minimize bias in the translation process. Variables such as continuous intention of binge-watching, habit, regret, perceived usefulness, and social influence were measured on a numerical scale (1 – strongly disagree, 7 – strongly agree). On the first page of the electronic questionnaire, respondents were informed about the objectives of the study, followed by the definition of binge-watching used for this study [1], which consists of the viewing of two or more episodes of the same show in just one sitting. Before continuing to the next page, the respondents had to accept the informed consent. Table 1 shows the measurement items and their respective sources.

**Table 1:** Measurement items and sources.

Construct	Source	Questions
Binge-Watching engagement	[11]	1. I feel caught up when I binge-watch.
		2. I feel focused when I binge-watch.
		3. Every time I binge-watch, I abstract myself from the rest around me.
Continuous intention of Binge-watching	[41]	1. I intend to continue binge-watching.
		2. I will always try to binge-watch in my daily life.
		3. I plan to continue binge-watching frequently.
Perceived usefulness	[27]	1. Binge-watching allows me to achieve relaxation and fun more quickly than other ways.
		2. Binge-watching allows me to achieve relaxation and fun effectively.
		3. Binge-watching allows me to satisfy relaxation and fun in an easier way than other ways.
Social Influence	[27]	1. My colleagues think that I should binge-watch.
		2. My classmates think that I should binge-watch.
		3. My friends think that I should binge-watch.
Habit	[41]	1. Binge-watching has become a habit for me.
		2. I binge-watch frequently.
		3. Binge-watching has become natural to me.
Regret	[42]	1. I wish I didn't binge-watch so much.
		2. I feel bad after binge-watching several episodes.
		3. I regret the real-life consequences of binge-watching (i.e., fewer hours of sleep, postponing chores, etc.)

Before the data collection, the electronic questionnaire was approved by the NOVA IMS Ethics Committee with ethical approval reference number 2021-4-23502, confirming that the study complies with all regulations required at the National and European levels. All participants agreed to voluntarily participate in the study, and informed consent was given by the participants in the electronic questionnaire.

### 4.2 Data analysis method

This investigation adopts the Partial Least Squares (PLS-SEM) method, a multivariate analysis approach to evaluate the theoretical model. PLS-SEM provides researchers the ability to integrate unobservable (latent) variables, which are measured indirectly through indicator variables, into their research frameworks, thus offering a more comprehensive understanding of the relationships among variables [43]. The minimum sample size to evaluate models using PLS-SEM follows the rule of thumb of having at least ten times the largest number of paths to a construct in the theoretical model. In our case, there are three paths directed to continuous intention to binge-watch.

The PLS approach serves especially when dealing with small sample sizes, data that do not conform to distributional assumptions, complex models, or situations where the primary motivation for modeling is exploratory in nature [43]. It is particularly advantageous in addressing research questions where traditional methods might struggle to produce meaningful results. To effectively apply the PLS-SEM technique to the current study, we utilized the SmartPLS 3 software [44].

### 4.3 Data collection

Due to the exploratory nature of this study and the unknown population size of the binge-watchers in Portugal, the use of a convenience sample seems appropriate in these circumstances. Convenience sampling has been successfully used in other studies that evaluated theoretical models that explain human behavior within technological and marketing contexts [45,46]. The link to the electronic questionnaire was shared via social media such as Instagram, Facebook, and LinkedIn from the 2nd to the April 30, 2021 using the snowball technique [47]. People were initially introduced to the topic of watching TV shows and asked to click on a hyperlink to the survey form if they identified themselves with that behavior. The authors collected a convenience sample of 225 valid responses, which is an appropriate sample size for evaluating theoretical models with PLS-SEM [48]. Table 2 presents the respondents’ profiles.

**Table 2:** Respondents’ profile.

Characteristics	n	%
Gender		
Male	97	43.11
Female	125	55.56
Other	2	0.89
Prefer not to say	1	0.44
Age		
<18	30	13.33
18–24	142	63.11
25–34	32	14.22
35–44	7	3.11
45–54	6	2.67
55–64	6	2.67
>65	1	0.44
Education		
Less than high school	4	1.78
High school	68	30.22
Bachelor's degree	94	41.78
Master's degree	50	22.22
Doctorate	5	2.22
Other	1	0.44

## 5 Results

### 5.1 Binge-watching measurement model results

The current model consists of six reflective constructs, which implies that all construct indicators are designed with the understanding that they measure the same underlying phenomenon (latent variable) [49]. To evaluate the internal consistency and reliability of reflective constructs, certain criteria must be satisfied. Assessing whether there are any multicollinearity issues or common method bias is done using the VIF values. Internal consistency is assessed through Cronbach's Alpha and Composite Reliability, both of which require construct values to be greater than 0.6 [50]. The model meets both of these criteria.

To check for multicollinearity and common method bias issues, the variance inflation factor (VIF) model was examined. All VIF values are below 5, indicating that the model has no collinearity issues [43]. Additionally, all VIF values in the inner model are below 3.3, which is the threshold suggested by Kock [51] to identify common method bias. Consequently, this indicates no common method bias issues in our model.

Convergent validity is evaluated using average variance extracted (AVE) and loadings. AVE measures the proportion of variance captured by a construct relative to the amount of variance due to measurement errors (see Table 3). To meet the convergent validity criteria, the AVE value must be greater than 0.5, and loadings must be greater than 0.7 [50]. The model satisfies both of these measures.

**Table 3:** Assessment of measurement model. Notes: SD = Standard Deviation, CA = Cronbach's Alfa, CR = Composite Reliability, AVE = Average Variance Extracted.

Constructs	Mean	SD	CA	CR	AVE
Binge-Watching Engagement	4.584	1.601	0.912	0.944	0.85
Continuous Intention of Binge-Watching	4.595	1.541	0.837	0.902	0.754
Habit	4.242	1.801	0.928	0.954	0.875
Perceived usefulness	4.474	1.441	0.885	0.929	0.813
Regret	3.543	1.648	0.837	0.9	0.75
Social Influence	3.02	1.494	0.802	0.883	0.716

Regarding discriminant validity, it is necessary to check if the measurement items measure the construct in question, i.e., to determine if the constructs are independent of one another or if they measure other (related) constructs. For that, the square root of the AVE of each construct was evaluated. The off-diagonal values shown in Table 4 represent the correlations within the constructs (i.e., Continuous Intention of Binge-Watching correlates with Binge-Watching Behavior at 0.437). On the diagonal, there are the values of the square root of the AVE. Following the Fornell & Larcker [52] criterion for discriminant validity, the square root of the AVE value must be greater than the values of intercorrelation. This criterion is met, meaning that each construct shares a higher degree of variance with its respective set of measures than with other constructs linked to distinct measure groups.

**Table 4:** Fornell-Larcker criterion.

Constructs	BWE	CIBW	H	PU	R	SI
Binge-Watching Engagement (BWE)	0.922					
Continuous Intention of Binge-Watching (CIBW)	0.437	0.869				
Habit (H)	0.48	0.626	0.935			
Perceived usefulness (PU)	0.473	0.607	0.529	0.901		
Regret (R)	0.107	−0.155	0.12	−0.045	0.866	
Social Influence (SI)	0.273	0.311	0.394	0.446	0.204	0.846

The Heterotrait-Monotrait criterion is also a discriminant validity measure that allows checking if the variables' measures diverge from each other. The values shown in Table 5 must be lower than 1, and as it is possible to verify, this criterion for discriminant validity is also met.

**Table 5:** Heterotrait-Monotrait Ratio (HTMT).

Constructs	BWE	CIBW	H	PU	R	SI
Binge-Watching Engagement (BWE)						
Continuous Intention of Binge-Watching (CIBW)	0.497					
Habit (H)	0.511	0.702				
Perceived usefulness (PU)	0.527	0.705	0.58			
Regret (R)	0.137	0.176	0.14	0.088		
Social Influence (SI)	0.327	0.381	0.457	0.522	0.254	

The cross-loadings criterion is also used to assess the discriminant validity of the scales used [49]. This criterion says that loading for the items in each construct must be greater than 0.7 and also greater than the cross-loadings. The loadings for each construct item are reported in Table 6. All the values meet the cross-loading criterion.

**Table 6:** Loadings and cross-loadings. Notes: BWE = Binge-Watching engagement, CIBW = Continuous Intention of Binge-Watching, H = Habit, PU = Perceived usefulness, R = Regret, SI = Social Influence.

Constructs	Item	BWE	CIBW	H	PU	R	SI
BWE	Engag01	0.946	0.423	0.499	0.44	0.093	0.235
	Engag02	0.948	0.431	0.463	0.471	0.061	0.254
	Engag03	0.869	0.343	0.347	0.394	0.155	0.274
CIBW	ContInt01	0.423	0.864	0.509	0.516	−0.207	0.246
	ContInt02	0.364	0.825	0.433	0.52	−0.062	0.303
	ContInt03	0.353	0.914	0.669	0.547	−0.129	0.268
H	Hab 01	0.421	0.576	0.933	0.469	0.108	0.344
	Hab 02	0.392	0.598	0.945	0.49	0.114	0.343
	Hab 03	0.525	0.582	0.928	0.521	0.114	0.414
PU	PercUse01	0.459	0.512	0.432	0.882	−0.059	0.349
	PercUse02	0.412	0.585	0.501	0.908	−0.089	0.399
	PercUse03	0.414	0.541	0.492	0.914	0.025	0.454
R	Regret 01	0.034	−0.144	0.177	−0.082	0.863	0.225
	Regret 02	0.115	−0.153	0.031	−0.021	0.908	0.146
	Regret 03	0.147	−0.093	0.114	−0.001	0.826	0.156
SI	SocInf01	0.276	0.242	0.332	0.343	0.237	0.867
	SocInf02	0.263	0.292	0.41	0.364	0.199	0.874
	SocInf03	0.162	0.254	0.264	0.415	0.094	0.796

### 5.2 Binge-watching structural model results

**Fig. 2:**
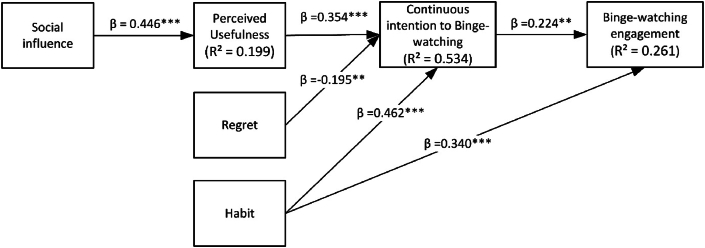
Theoretical Model including path coefficients. Note: *p < 0.1, **p < 0.05, ***p < 0.001.

To evaluate the structural model and empirically validate the hypotheses, path coefficients and variance explained were examined. The bootstrapping procedure was used with a subsample of 5000 samples. The three latent variables (Regret, Perceived usefulness, and Habit) explain 53.4% of the variance in the Continuous intention of binge-watching, and the two variables (Habit and Continuous intention of binge-watching) explain 26.1% of the variance in binge-watching behavior. Social influence explains 19.9% of the variance in Perceived usefulness. Please see Fig. 2 for the structural model results.

All the path coefficients were significant, as hypothesized. Habit has the strongest effect on Binge-watching Behavior (β = 0.340; ρ < 0.01), followed by Continuous Intention of binge-watching (β = 0.224 ρ < 0.05). These path coefficients support, respectively, hypotheses H6 and H3. Habit has the strongest effect on Continuous Intention of binge-watching (β = 0.462; ρ < 0.01), followed by Perceived Usefulness (β = 0.354; ρ < 0.01) and Regret, which, unlike the others, has a negative effect on it (β = −0.195; ρ < 0.05). Thus, these values support hypotheses H5, H2, and H4, respectively. The hypothesized relationship between Social Influence and Perceived Usefulness was also statistically significant (β = 0.446; ρ < 0.01), supporting hypothesis H1.

Lastly, an indirect effect of Social Influence through Perceived Usefulness on the Continuous Intention of Binge-Watching was found. The values concerning the specific indirect effects of the model indicate that the path coefficient for the indirect effect of Social Influence on the Continuous Intention of binge-watching through Perceived Usefulness was significant (β = 0.158; ρ < 0.01). The results are summarized in Table 7.

**Table 7:** Results of hypotheses assessment.

Hypotheses	Independent Variable	Dependent Variable	Path coefficient	Results
H1	Social Influence	Perceived Usefulness	(β = 0.446; ρ < 0.01)	Supported
H2	Perceived Usefulness	Continuous Intention of Binge-Watching	(β = 0.354; ρ < 0.01)	Supported
H3	Continuous Intention of Binge-Watching	Binge-Watching Engagement	(β = 0.224; ρ < 0.05)	Supported
H4	Regret	Continuous Intention of Binge-Watching	(β = −0.195; ρ < 0.05)	Supported
H5	Habit	Continuous Intention of Binge-Watching	(β = 0.462; ρ < 0.01)	Supported
H6	Habit	Binge-Watching Engagement	(β = 0.340; ρ < 0.01)	Supported

The model was also evaluated using a multigroup analysis technique in SmartPLS to assess individual differences by gender. The main difference between females and males is that continuous intention was statistically significant only for males, and regret over continuous intention was statistically significant only for females. The rest of the hypotheses were statistically significant for females and males. The results of the multigroup analysis by gender are presented in Table 8.

**Table 8:** Differences in the model evaluation by females and males. Note: *p < 0.1, **p < 0.05, ***p < 0.001.

Hypotheses	Independent variable	Dependent variable	Beta(female)	Beta (male)	Difference (female-male)
H1	Social Influence	Perceive Usefulness	0.480***	0.415***	0.064
H2	Perceive Usefulness	Continuous Intention	0.280***	0.457***	−0.177
H3	Continuous Intention	Binge-Watching Engagement	0.097	0.400***	−0.303**
H4	Regret	Continuous Intention	−0.259***	−0.098	−0.162
H5	Habit	Continuous Intention	0.500***	0.404***	0.096
H6	Habit	Binge-Watching Engagement	0.331***	0.288***	0.043

## 6 Discussion

The results demonstrate that the continuous intention of binge-watching significantly influences engagement in the binge-watching, being this effect particularly stronger for males. However, the intention to binge-watch, although impactful, is a weaker predictor of binge-watching engagement than habit. This finding aligns with the notion that habits can make intentions a less reliable predictor of behavior [37]. Furthermore, Kim et al. [53] suggested that the intention-usage relationship is generally weaker among heavier users than lighter users, indicating that stronger past use weakens the predictive power of intentions. This could be explained by the fact that individuals who develop a habit tend to engage in that behavior more automatically rather than deliberately. People often perform habitual actions in response to environmental cues rather than through conscious deliberation. This explains why the presence of habit has a stronger effect on binge-watching engagement than intention. Furthermore, individuals might consider reducing their binge-watching behavior due to feelings of regret but ultimately continue engaging in it due to habit.

Feelings of regret after binge-watching can lead to a reduced intention to continue the behavior, consequently lessening the influence of intention on binge-watching engagement [16,21,36]. The regret construct aligns with previous studies on binge-watching, confirming that excessive engagement often triggers negative emotions. Interestingly, when the model was evaluated separately for females and males, regret over continuous intention was statistically significant only for females. However, further research is needed to draw final conclusions about these differences by gender.

Perceived usefulness was found to have a substantial impact on the intention of binge-watching, ranking as the second strongest predictor after habit. This suggests that the existence of habit is more crucial than an individual's perception of binge-watching as an effective form of entertainment when forming intentions to continue the behavior. A possible explanation is that people initially start binge-watching to alleviate boredom and occupy their time [12,54], which subsequently evolves into a habit.

As hypothesized, social influence positively impacts perceived usefulness, consistent with previous studies investigating similar connections [[23], [24], [25]]. The results confirm that when friends, family members, or colleagues recommend binge-watching a TV series, it influences one's perception of the activity through the process of internalization. Furthermore, binge-watching the same series as others in one's social circle may foster additional social interactions. Social influence, operating through internalization and identification, relates to altering belief structures and potentially elevating one's status in the eyes of others. The study found that social influence indirectly affected binge-watching intention through perceived usefulness, indicating that when others perceive binge-watching as enjoyable entertainment, individuals are more likely to adopt the same belief.

### 6.1 Theoretical implications

This study provides three theoretical implications. First, binge-watching is a relatively recent phenomenon, although widespread, that has been little explored in the literature. Motivations of binge-watching have been studied, such as enjoyment, efficiency, recommendation of others, perceived control, fandom, sense of efficiency, engagement, entertainment, relaxation, boredom relief, and escapism [1,11,12,14]. Our study sheds more light on the drivers of binge-watching continuous intention and engagement behavior.

Second, even though previous studies have shown that in the presence of habit, defined by the extent to which a person perceives the behavior to be automatic, the intention is no longer relevant [37] to explain behavior. Our study provides evidence that in the context of binge-watching, habit does not eclipse continuous intention and that both are relevant to explaining binge-watching engaging behavior.

Third, previous studies have examined regret as a consequence of binge-watching [16,36]. Our study confirms previous findings in the sense that it is usual for binge-watchers to experience a sense of regret after binge-watching. However, different from previous studies, we explore the negative association between regret and continuous intention. Consequently, showing that people who experience these feelings of regret are likely to diminish their intention to continue binge-watching but not stop them from doing it.

### 6.2 Practical implications

Our findings are relevant to the psychological field in the sense that they reveal new findings about how binge-watching can be greatly influenced by the presence of a habit (how people perceive the act of binge-watching as something automatic to themselves) and the intention of continuing to do it, possibly revealing that people can be doing it just because they are used to it and not because they really want to. We also found that many people experience feelings of regret after a binge-watching session, which consequently lowers their intention to continue doing it. Furthermore, habit turned out to be a stronger influence than the intention over the behavior, which perhaps points to signs of addiction [[55], [56], [57]].

Results showing that there are people experiencing feelings of regret can be valuable for streaming platforms [58] that seek to provide their customers with options to make them feel less guilty about binge-watching. Thus creating a healthy binge-watching culture and content consumption. Providers may wish to consider adding features that remind viewers of how much time they have already spent watching and let them set their own limits. In this way, people might feel safer and continue consuming content from the streaming platform instead of feeling tempted to stop binge-watching.

## 7 Conclusion

This study contributes to our understanding of binge-watching behavior by examining the roles of habit, continuous intention, and feelings of regret in predicting engagement in this increasingly prevalent form of media consumption. The findings reveal that habit is a more potent predictor of binge-watching engagement than intention, suggesting that habitual behaviors may overshadow conscious intentions in this context. This insight aligns with previous research indicating that habits can make intentions less reliable predictors of behavior and that individuals who develop habits tend to engage in behaviors more automatically rather than deliberately. Furthermore, the study demonstrates that feelings of regret after binge-watching negatively influence continuous intention yet do not necessarily lead to a complete cessation of the behavior.

These findings shed light on the complex interplay between emotions and behavioral intentions in the context of binge-watching. Additionally, the study highlights the significant impact of perceived usefulness and social influence on the intention to binge-watch. These insights contribute to the existing literature on binge-watching and hold practical relevance for mental health professionals, streaming platform providers, and policymakers aiming to promote healthier media consumption habits. Our findings emphasize the need to consider the role of habit formation in developing effective strategies to mitigate the potential negative consequences of binge-watching on mental health and well-being. Streaming platforms could implement features that remind viewers of the time they spend watching and allow them to set personal limits, fostering a more mindful and guilt-free content consumption culture.

### 7.1 Limitations and future research

Several limitations should be taken into consideration for future studies. First, the generalization of the findings to the general population is questionable since the theoretical model of the present study was tested on a sample of a single European country population, which may not represent a comprehensive universe of binge-watchers. Second, our sample was limited in terms of age range, with most (63.11%) of the respondents between the ages of 18 and 24. This may not represent the binge-watchers population as a whole in the best way since people of this age range can have lifestyles that are very different from those of older and younger viewers and thus have different binge-watching behaviors and intentions [59]. Third, this is a cross-sectional study, which may limit inferring the direction of the causation. And fourth, some potential drivers may be missing from the model, or some constructs may need evaluation with different conceptualization in the model, for instance, binge-watching engagement associated with device overuse or addiction, or regret, that is hypothesized as negative driver for continuous intention could be evaluated as consequence of binge-watching too. Future research may adjust the model to include different drivers and compare the results with the model evaluated in this study. Furthermore, new studies may follow a longitudinal approach with a larger and more diverse international sample to diminish these limitations and enhance generalizability. For future research, it would also be important to assess the possible differences in behavior engagement regarding the ages of individuals, as well as if the continuous intention behavior and engagement differ from the various genres of content in VOD.

## Data availability statement

Data is available upon request.

## Ethics declarations

The electronic questionnaire was approved by the NOVA IMS Ethics Committee with ethical approval reference number 2021-4-23502. All participants provided informed consent to participate in the study.

## Consent to publish

All of the authors consented to publish this manuscript.

## Funding

We gratefully acknowledge financial support from 10.13039/501100001871FCT Fundação para a Ciência e a Tecnologia (Portugal), national funding through research grant Information Management Research Center – MagIC/10.13039/501100005855NOVA IMS (UIDB/04152/2020).

## CRediT authorship contribution statement

**Maria Bastos:** Writing – review & editing, Writing – original draft, Methodology, Investigation, Formal analysis, Data curation, Conceptualization. **Mijail Naranjo-Zolotov:** Writing – review & editing, Validation, Supervision, Methodology, Funding acquisition, Formal analysis. **Manuela Aparício:** Writing – review & editing, Validation, Funding acquisition.

## Declaration of competing interest

The authors declare that they have no known competing financial interests or personal relationships that could have appeared to influence the work reported in this paper.
