# Supplementary material for: Binge-watching Uncovered: Examining the interplay of perceived usefulness, habit, and regret in continuous viewing
Source: Heliyon. 2024 Mar 13;10(6):e27848. doi: 10.1016/j.heliyon.2024.e27848 (PMC10955278; doi:10.1016/j.heliyon.2024.e27848)
Supplement: Multimedia component 1 [file mmc1.docx]

Supplementary Material - Questionnaire

Please answer the following questions on a numerical range scale from 1 – strongly disagree to 7 – strongly agree.

| Constructs | Questions |
| --- | --- |
| Binge-Watching engagement | 1. I feel caught up when I binge-watch. |
|  | 2. I feel focused when I binge-watch. |
|  | 3. Every time I binge-watch, I abstract myself from the rest around me. |
| Continuous intention of Binge-watching | 1. I intend to continue binge-watching. |
|  | 2. I will always try to binge-watch in my daily life. |
|  | 3. I plan to continue binge-watching frequently. |
| Perceived usefulness | 1. Binge-watching allows me to achieve relaxation, fun, more quickly than other ways. |
|  | 2. Binge-watching allows me to achieve relaxation, fun, effectively. |
|  | 3. Binge-watching allows me to satisfy relaxation, fun, in an easier way than other ways. |
| Social Influence | 1. My colleagues think that I should binge-watch. |
|  | 2. My classmates think that I should binge-watch. |
|  | 3. My friends think that I should binge-watch. |
| Habit | 1. Binge-watching has become a habit for me. |
|  | 2. I binge-watch frequently. |
|  | 3. Binge-watching has become natural to me. |
| Regret | 1. I wish I didn’t binge-watch so much. |
|  | 2. I feel bad after binge-watching several episodes. |
|  | 3. I regret the real-life consequences of binge-watching (i.e. fewer hours of sleep, postponing chores, etc.) |

Demographic questions

| Please indicate your gender | |
| --- | --- |
| Male |  |
| Female |  |
| Other |  |
| Prefer not to say |  |
| Please indicate your age | |
| <18 |  |
| 18-24 |  |
| 25-34 |  |
| 35-44 |  |
| 45-54 |  |
| 55-64 |  |
| >65 |  |
| Please indicate your education level | |
| Less than high school |  |
| High school |  |
| Bachelor's degree |  |
| Master's degree |  |
| Doctorate |  |
| Other |  |
